# Structural insight into polyphenol oxidation during black tea fermentation

**DOI:** 10.1016/j.fochx.2023.100615

**Published:** 2023-02-26

**Authors:** Lin Chen, Huajie Wang, Yang Ye, Yuefei Wang, Ping Xu

**Affiliations:** a Institute of Tea Science, Zhejiang University, Hangzhou 310058, China; b Tea Research Institute, Chinese Academy of Agricultural Sciences, Hangzhou 310008, China; c Key Laboratory of Horticultural Plant Growth, Development and Quality Improvement, Ministry of Agriculture, Hangzhou 310058, China

**Keywords:** Black tea, Tea polyphenols, Oxidation kinetic, Molecular structure, Fermentation

## Abstract

Polyphenol oxidation during fermentation plays a critical role in the formation of flavor and function of black tea. However, how the specific structures of tea polyphenols affect their oxidation kinetics during black tea fermentation is still unknown. Here, we found that the oxidations of tea polyphenols, including 7 catechins, 4 phenolic acids and 11 flavonoid glycosides followed pseudo-first-order kinetics during fermentation. Molecular structure and oxygen concentration collaboratively regulated the oxidation rate of different polyphenols. Pyrogallol structure was more easily to be oxidized than catechol and monophenol structure in B-ring, the gallic group in C-ring could inhibit oxidation of catechins, while the role of sugar moiety of flavonoid glycosides was differentiating. In addition, oxygen was found to be the key factor limiting the oxidation rate of polyphenols in regular black tea fermentation, and the oxidation rate constants of tea polyphenols were linearly and positively correlated with oxygen concentration.

## Highlights

• Polyphenol oxidation followed pseudo-first-order kinetic during fermentation.
• Oxidation rate of structure in B-ring: pyrogallol > catechol > monophenol.
• Gallic group and sugar moiety in C-ring affect polyphenol oxidation.
• Polyphenol oxidation rate was linearly correlated with oxygen concentration.
• Oxygen, not enzyme, is the factor limiting the oxidation rate of most polyphenols.

## 1 Introduction

Polyphenols are the key compounds contributing to the flavor quality and function properties of tea leaf-derived products. The degree of polyphenol oxidation during processing is the criterion for classifying tea into six tea types (Meng et al., 2019, Li et al., 2022). Among them, black tea is the most produced and consumed tea worldwide and the manufacturing process mainly includes withering, rolling, fermentation, and drying, where fermentation is considered as the critical step for the substance transformation and quality formation. Many reactions take place simultaneously in black tea fermentation, including oxidation of polyphenols, conversion of amino acids, and fading and formation of aroma metabolites, which shape the unique vibrant red color, mellow, sweet, and umami taste, and floral or fruity like aroma of black tea (Wang et al., 2023, Liu et al., 2023). As known, polyphenol oxidation catalyzed by polyphenol oxidase (PPO) and peroxidase (POD) is the core reaction in black tea fermentation (Tan et al., 2016, Guo et al., 2021). Polyphenols can be oxidized and polymerized by themselves to form oxidation products such as theaflavins (TFs), theasinensins (TSs) and thearubigins (TRs), and also couple with amino acid degradation to form volatile aldehyde metabolites, which improve the aroma and taste of black tea (Chen, et al., 2021). Thus, understanding the polyphenols oxidation kinetics is helpful for modifying the flavor quality and function properties of black tea by regulating fermentation conditions.

Polyphenols in fresh tea leave mainly includes catechins, flavonols and flavonoid glycosides, anthocyanins, and phenolic acids according to the structure features. Except for phenolic acids, the polyphenols are all the derivatives of benzopyran or chromane, which have the basic skeleton of C6-C3-C6 configuration (Truong and Jeong, 2022). Catechins, including (−)-epigallocatechin gallate (EGCG), gallocatechin gallate (GCG), (−)-epigallocatechin (EGC), gallocatechin (GC), (−)-epicatechin (EC), catechin (C), (−)-epicatechin gallate (ECG), and catechin gallate (CG), are the dominant polyphenols in raw tea leaves (Scharbert et al., 2004, Stodt et al., 2014, Abd-Elsalam et al., 2014). According to the configuration of the two hydrogens at the 2nd and 3rd position in the C-ring, catechins could be divided into *cis* and *trans* forms (Sirk, Brown, Sum, & Friedman, 2008), and according to the amount of hydroxyl groups in B-ring, they can also be divided into pyrogallol form and catechol form. Besides, ester catechins have a gallate group attached to the C-ring at the 3-position compared to non-ester catechins. Flavonoid glycosides are important taste components and are made from aglycone kaempferol, quercetin, myricetin conjugated with sugar moiety glucose, galactose, rhamnose, and arabinose, and usually consist of mono-, di- and tri-glycosides (Dai et al., 2017, Chen et al., 2021). The amount of hydroxyl groups in B-ring and the type of sugar moiety in C-ring at 3-position is the main difference between flavonoid glycosides in tea leaves. However, the potential impact of molecular structures of polyphenols on their oxidation during fermentation is still elusive.

Besides, oxygen is an important substrate of the oxidation reaction. Increasing oxygen can promote the consumption of catechins in fermentation (Pan, Shen, Shen, & Du, 2014). And our previous work showed that polyphenols, including catechins, phenolic acids, and flavonoid glycosides decreased by 10–30% in high oxygen fermentation group (35%) compared to nature fermentation group (21%) by promoting oxidation conversion (Chen, et al., 2021). The flow rate of oxygen during the suspension fermentation of tea fresh leaves could affect the ratio of oxidation products, like TFs and TSs, attributed to the different oxidation pathways (Xu, Jiang, Xue, Liu, Zhang, & Wang, 2014). However, the specific role of oxygen on the oxidation of polyphenols with different molecular structures during black tea fermentation is not clear.

Therefore, in the present work, the oxidation kinetics of polyphenols including catechins, flavonoid glycosides and phenolic acids during black tea fermentation were investigated by employing a non-targeted metabolomics, meanwhile, the effect of molecular structures of polyphenols on their oxidations were deeply explored. Furthermore, fermentation with different oxygen concentrations were performed to reveal the potential impact of oxygen on polyphenols oxidation with particular structures.

## 2 Materials and methods

### 2.1 Chemicals and instruments

The tea processing instruments withering room, rollers (6CR-35), and fermentation platform was all provided by Tea Research Institute, Chinese Academy of Agricultural Science. The withering room can regulate the environment temperature and humidity and the fermentation platform can regulate the temperature, humidity and oxygen concentration. The ES108-LCD oxygen detectors were purchased from Hangzhou Jiachang electronic science and technology company (China). Methanol, acetonitrile, formic acid for UPLC-MS spectrometry purchased from Fisher Scientific (USA).

### 2.2 Tea sample preparation

According to our previous research to make tea samples, the method is shown in Fig. S1 (Chen, et al., 2021). The fresh tea leaves were picked from Longjing 43, which were planted in the Shengzhou, Shaoxing city of Zhejiang province (120.83**°**N, 29.74**°**E), and the standard of tea was a bud with two leaves. After picking, tea leaves were immediately wilted and the temperature and relative humidity of the withering room were controlled to 28 °C and 65% respectively. The tea leaves moisture content dropped from 77% to 60.22% after withering. Subsequently, the withered leaves were rolled for 70 mins by 6CR-35 roller. And then the rolled leaves were equally divided into 3 portions for fermentation separately in tea fermentation platform with environment temperature 30 °C and a relative humidity of 90%.

Three oxygen concentration groups were regulated for black tea fermentation to research the role of oxygen concentration on the degradation kinetics of polyphenols. The oxygen concentration was measured in real time through the ES108-LCD oxygen detectors. The oxygen concentration of different groups was 5% (L), 21% (CK), and 35% (H), respectively. The oxygen concentrations of groups L and H were within 5% of error. The L group decreased the oxygen concentration by uniformly delivering nitrogen to the fermentation equipment through the nitrogen tank. The H group increased the oxygen concentration by uniformly delivering oxygen to the fermentation equipment through the oxygen tank. The CK group was naturally fermented using oxygen in the air. Fermentation time of 3 groups was all 5 h. Tea samples were taken at 1 h intervals in the 3 groups and stored rapidly in liquid nitrogen and then were freeze-dried and then stored in −20 °C refrigerator for detection.

### 2.3 Detection method of polyphenols

The extraction and detection method of polyphenols was described in our previous article (Chen, et al., 2021). A 200 mg of freeze-dried ground tea powder was extracted with 10 mL 70% methanol. The extraction time was 10 min and temperature were 70 °C. The extract solution was filtered at 0.22 µm membrane after centrifuging at 3500 r/min for 10 min.

Polyphenols were analyzed using the method reported in our previous article by IPhenome biotechnology (Yun Pu Kang) Inc. Dalian, China (Chen, et al., 2021). The UPLC-HRMS system was an Ultimate 3000 separation module coupled with Q-Exactive mass spectrometer. The column used was a 2.1 × 100 mm, 1.8 μm, HSS T3 purchased from Waters. Mobile phase A (distilled water and 0.1% formic acid) and mobile phase B (acetonitrile and 0.1% formic acid), were used as eluant. The linear gradient of the detection method ramped from 5% mobile phase A to 90% in 20 min. Mass spectrometry conditions: positive/negative ionization with a scan range of 100 ∼ 1000 *m*/*z*, aux gas was 10 arb, heater temperature was 355 °C, capillary temperature was 320 °C, the sheath gas was 45 arb, and S-Lens RF level was 55%.

### 2.4 Oxidation kinetics

Our previous study revealed that the catechins degradation in aqueous solution at pH 5.6 with different temperature followed apparent first-order kinetics (Chen, Wang, Zhang, Cui, Ni & Jiang, 2021). And our previous study defined degradation as all reactions that cause loss of catechins, including oxidation and epimerization. Although, much more complex mechanisms were involved during black tea fermentation, the core reaction of polyphenols during fermentation was oxidation. Thus, pseudo-first-order kinetic models could be applied to the concentration of polyphenols over fermentation time to compare the stability of polyphenols. And the R^2^ value can be used to assess the accuracy of the model. Therefore, the concentration of polyphenols was revealed by the following equation:

Ct/C0=exp(-kt)

C0 and Ct in the equation represent the concentration of polyphenols at time 0 and t, respectively, and k is the rate constant. Thus, in the case of ln (Ct/C0) with time, the k value of polyphenol degradation can be calculated from the best-fitting linear gradient.

### 2.5 Statistical analysis

The peak table of different polyphenols were analyzed by Office Excel software (Microsoft, USA). The significance tests were adopted at *P* < 0.005 and performed with SPASS 19.0; The histogram and heat map drawn by Origin 2022. And hierarchical clustering analysis and linear fitting was generated using Origin 2022.

## 3 Results

### 3.1 Characterization of polyphenol during black tea fermentation

**Fig. 1:**
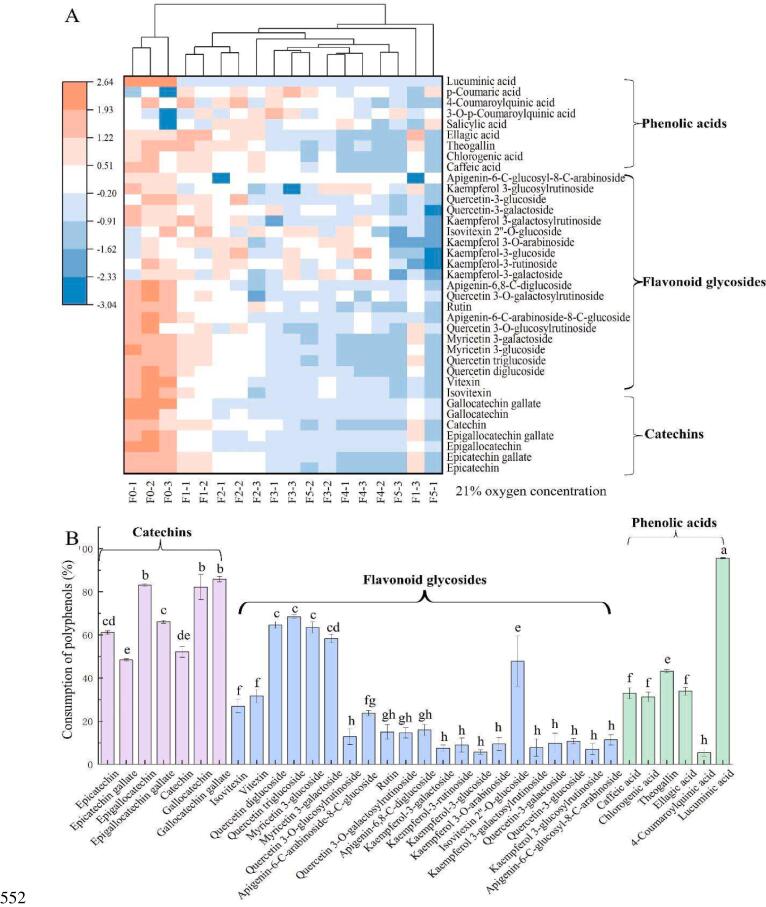
Heat map of the changes of different polyphenols during the fermentation of black tea (A); Consumption ratio of polyphenols with significant differences at the end of fermentation (B).

The non-targeted metabolomics resultes showed that a total of 37 polyphenols, including 7 catechins, 21 flavonoid glycosides, 9 phenolic acids, were obtained after structural identification. The changes of polyphenols during black tea fermentation is shown in Fig. 1(A). It can be seen that the content of most of the polyphenols tended to decrease as the fermentation time increased. Analysis of variance (ANOVA) showed that all polyphenols, except 3 phenolic acids (salicylic acid, 3-O-p-coumaroylquinic acid, and p-coumaric acid) were significantly decreased (*P* < 0.05). Furthermore, the consumption ratio of polyphenols with different structures varied greatly in black tea fermentation as shown in Fig. 1(B). It can be observed that all catechins were decreased by above 50%, where GCG was with the highest consumption of 85%. The consumption ratio of flavonoid glycosides ranged from 5% to 68%, with great variation among different structures. The consumoption ratio of phenolic acids, expect for 4-coumaroylquinic acid and lucuminic acid, ranged from 31% to 43%.

### 3.2 Effects of molecular structure on polyphenols oxidation during black tea fermentation

In order to clarify the specific oxidation kinetics of polyphenols during fermentation, a pseudo-first-order kinetic equation was used to fit the variation of polyphenol content. The rate constant k value of the pseudo-first-order kinetics for oxidation of polyphenols during fermentation were calculated from plotting ln (Ct/C0) vs time. A total of 22 polyphenols, including 7 catechins, 4 phenolic acids, and 11 flavonoid glycosides, fitted a pseudo-first-order reaction with a coefficient of correlation R^2^ > 0.85 (Table S1). The high values of R^2^ confirmed the hypothesis that polyphenol oxidation followed pseudo-first-order kinetics during fermentation.

#### 3.2.1 Oxidation kinetics of catechins during fermentation

**Fig. 2:**
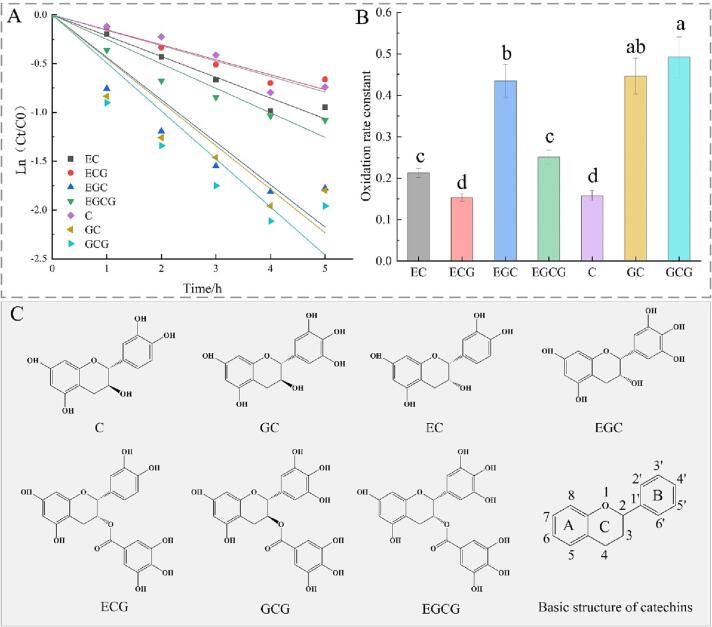
Effect of molecular structure of catchins on the kinetcis of oxidation: (A) Pseudo-first-order kinetics of different catechins oxidation; (B) Oxidation rate constant of catechins; (C) Molecular structure of different catechins.

As described above, a total of 7 catechins were found to follow a pseudo-first-order kinetic during black tea fermentation. The pseudo-first-order kinetic curves of catechins oxidation and the oxidation rate constants (k values) are shown in Fig. 2(A) and (B), respectively. The k value of EC, ECG, EGC, EGCG, C, GC, and GCG oxidation were found to be 0.2131, 0.15343, 0.43507, 0.25086, 0.15802, 0.44657, and 0.49221, respectively. Obviously, GCG, GC and EGC had the fastest oxidation rate with k values>0.4, followed by EGCG and EC with k values above 0.2, and ECG and EC degraded slowest with k values about 0.15. The oxidation rate constants k of the 7 catechins are, in descending: GCG > GC > EGC > EGCG > EC > C > ECG.

To verify the potential role of molecular structure on the catechins stability, the oxidation rates of different catechins were compared with each other. The K value of EGCG, EGC and GC was significantly (*P* < 0.05) higher than that of ECG, EC and C, respectively. Combined with the structure of catechins in Fig. 2(C), it can be seen that three adjacent hydroxyl groups at positions 3′-C, 4′-C, and 5′-C in the B ring were more easily oxidized compared with the structure of two adjacent hydroxyl groups at 3′-C and 4′-C. The k value of EC and EGC was significantly (*P* < 0.05) higher than that of ECG and EGCG, respectively. And the k value of GC and GCG had no significant difference, which indicated that the gallic group in C-ring was not a major factor affecting the degradation rate of catechins, but might limit the oxidation of catechins, especially those with a *cis*-structure.

#### 3.2.2 Oxidation kinetics of flavonoid glycosides during fermentation

**Fig. 3:**
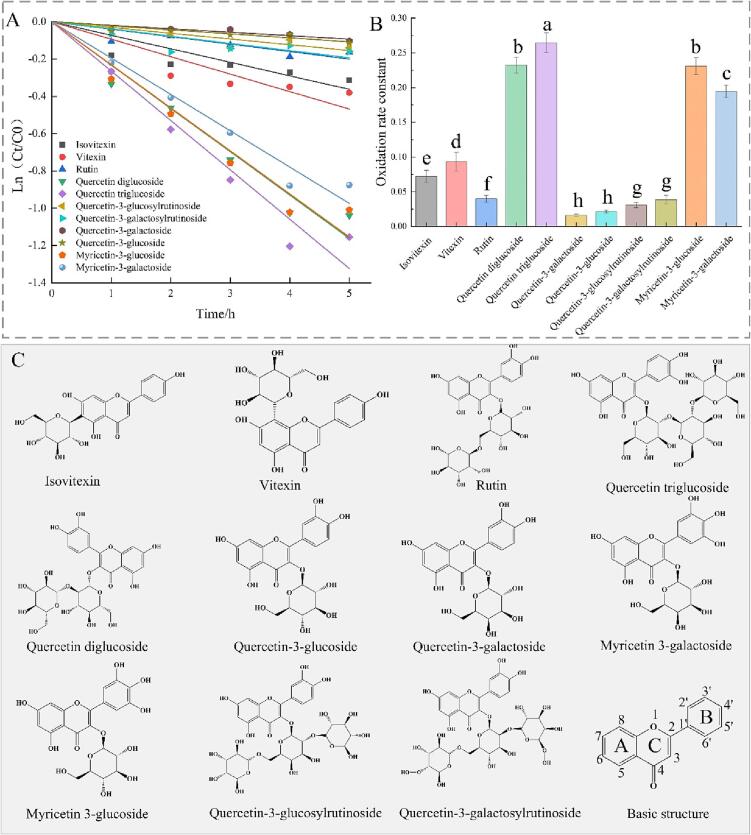
Effect of molecular structure of flavonoid glycosides on the kinetcis of oxidation: (A) Pseudo-first-order kinetics of different flavonoid glycosides oxidation; (B) Oxidation rate constant of flavonoid glycosides; (C) Molecular structure of different flavonoid glycosides.

A total of 11 flavonoid glycosides were observed to fit to the pseudo-first-order kinetic with R^2^ value > 0.85 during fermentation. The Fig. 3(A) and (B) showed the pseudo-first-order kinetic curves of flavonoid glycoside oxidation and the oxidation rate constants (k values), respectively. The oxidation rate constants of isovitexin, vitexin, rutin, quercetin diglucoside, quercetin triglucoside, quercetin-3-galactoside, quercetin-3-glucoside, quercetin-3-glucosylrutinoside, quercetin-3-galactosylrutinoside, myricetin-3-glucoside, and myricetin-3-galactoside were 0.07246, 0.0936, 0.03999, 0.23226, 0.26472, 0.01603, 0.02161, 0.0308, 0.03863, 0.23088, and 0.19479, respectively. The oxidation kinetics of the 11 flavonoid glycosides therefore decreased in the following order: quercetin triglucoside > quercetin diglucoside > myricetin 3-glucoside > myricetin 3-galactoside > vitexin > isovitexin > rutin > quercetin-3-galactosylrutinoside > quercetin-3-glucosylrutinoside > quercetin-3-glucoside > quercetin-3-galactoside.

As exhibited in Fig. 3(B), the k value of myricetin 3-glucoside was significantly higher than that of quercetin-3-glucoside, and k value of myricetin-3-galactoside was significantly higher than that of quercetin-3-galactoside. Since the R^2^ values of the pseudo-first-order kinetic equations fitted for kaempferol glycoside oxidation were not high, the consumption ratio in Fig. 1(B) was used for the comparison. The consumption ratio of kaempferol-3-galactoside, kaempferol-3-glucoside, quercetin-3-galactoside, quercetin-3-glucoside, myricetin-3-galactoside and myricetin-3-glucoside were found to be 7.56%, 5.76%, 9.81%, 10.74%, 58.29%, and 63.45%, respectively. The consumption of kaempferol glycosides had no significant (*P* < 0.05) difference with that of quercetin glycosides, while myricetin glycosides were significantly higher than that of kaempferol and quercetin glycosides. It can be seen that pyrogallol structure in B-ring of flavonoid glycosides was more unstable during fermentation than the catechol and monophenol structure in the B-ring.

In terms of k values, myricetin-3-glucoside was significantly higher than myricetin-3-galactoside, and quercetin triglucoside was significantly higher than quercetin diglucoside, quercetin triglucoside and quercetin diglucoside were significantly higher than quercetin-3-glucoside and quercetin-3-galactoside, which implied that the sugar moiety could affect the oxidation rate of flavonoid glycosides.

#### 3.2.3 Oxidation kinetics of phenolic acids during fermentation

**Fig. 4:**
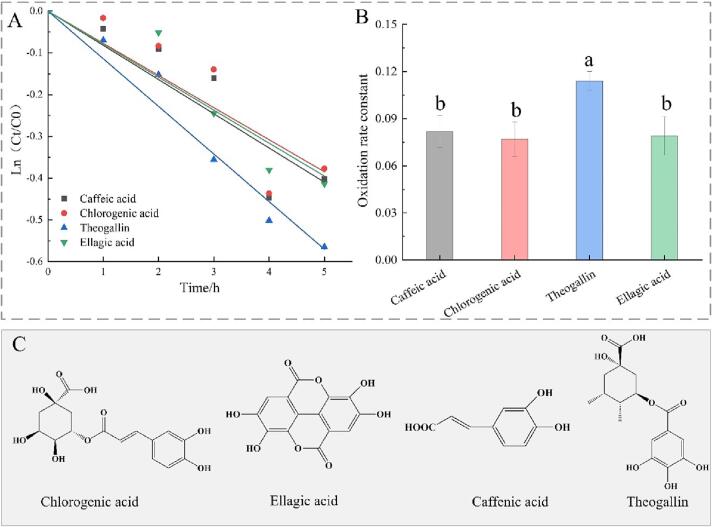
Effect of molecular structure of phenolic acids on the kinetcis of oxidation: (A) Pseudo-first-order kinetics of different phenolic acids oxidation; (B) Oxidation rate constant of phenolic acids; (C) Molecular structure of different phenolic acids.

Four phenolic acids were found to fit to the pseudo-first-order kinetic with R^2^ value > 0.85 during fermentation. The pseudo-first-order kinetic curves of phenolic acids oxidation and the oxidation rate constants k are shown in Fig. 4(A) and (B), respectively. The oxidation rate constants of caffeic acid, chlorogenic acid, theogallin, and ellagic acid were 0.08188, 0.07901, 0.11402, and 0.07246, respectively. The oxidation rate constants k of the 4 phenolic acids are, in descending: theogallin > caffeic acid > chlorogenic acid > ellagic acid. The k value of theogallin was significantly higher than that of others, while the k values of caffeic acid, chlorogenic acid, and ellagic acid had no significant difference. Combined with the structure of phenolic acids in Fig. 4(C), it can be seen that the pyrogallol structure of phenolic acids was more likely to be oxidized compared with catechol structure.

#### 3.2.4 Oxidation kinetics of different groups of polyphenols

The oxidation rate constants of three groups of catechins, flavonoid glycosides and phenolic acids were compared in Fig. S1. As exhibited in Fig. S2(B), the k values of GCG, GC, and EGC were much higher than those of other polyphenols. The k values of quercetin triglucoside, quercetin diglucoside, and myricetin-3-glucoside were not significantly different (*P* > 0.05) from those of EGCG and EC, and k value of myricetin-3-galactoside was not significantly (*P* > 0.05) different from that of EC. The k values of four phenolic acids had no significant difference (*P* > 0.05) with those of vitexin, and isovitexin. Four quercetin glycosides had the lowest oxidation rate constants. It can be seen that the average k values of 7 catechins, 11 flavonoid glycosides and 4 phenolic acids were 0.25086, 0.07264 and 0.08045, respectively, implying that catechin oxidation was faster than flavonoid glycosides and phenolic acids during black tea fermentation.

### 3.3 Effect of oxygen on different polyphenols oxidation kinetics during fermentation

**Fig. 5:**
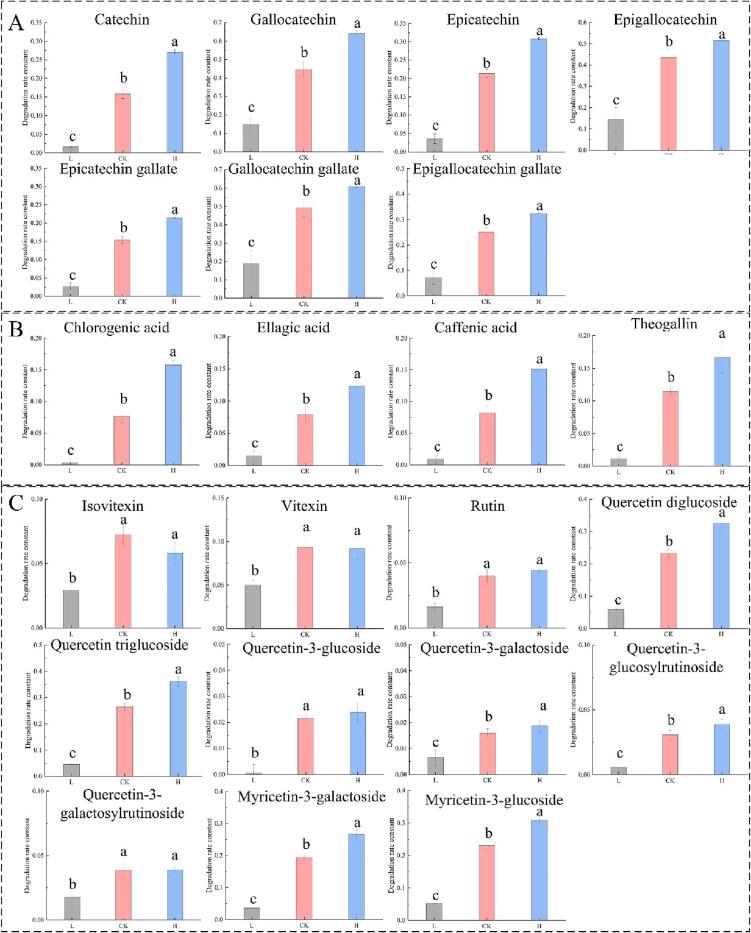
Effect of oxygen concentration on the oxidation kinetics of different polyphenols during fermentation flavonoid glycosides: (A) Catechins; (B) Phenolic acids; (C) Flavonoid glycosides.

The changes of the oxidation rate constant k of polyphenols under different oxygen concentrations are shown in Fig. 5. As shown in Fig. 5(A), the k values of catechins gradually increased with increasing oxygen. And the rate constants (k values) of EC, ECG, EGC, EGCG, C, GC, and GCG in H group increased 44.66%, 39.74%, 18.41%, 28.97%, 71.54%, 43.87%, and 23.73% compared with the CK group, respectively. Interestingly, a linear correlation with R^2^ > 0.95 was found between the oxygen concentrations and the k values, which meant oxygen concentration could be the determining or limiting factor for the oxidation rate of catechins. The linear fitted curves as well as the slope (k_2_) and R^2^ values are shown in Fig. S2 and Table S2, respectively. The k_2_ values of EC, ECG, EGC, EGCG, C, GC, and GCG were 0.91323, 0.64228, 1.64904, 1.00233, 0.76204, 1.92803, and 1.92766, respectively. It was found that the slope values of EGC, EGCG, GC, and GCG were higher than those of EC, ECG, and C, which implied that the pyrogallol structure was more sensitive to oxygen than the catechol structure. The k_2_ of ECG was lower than that of EC, and which of EGCG was lower than that of EGC, indicating that gallic groups had negativity effect on the catechins oxidation.

Meanwhile, as shown in Fig. 5(B), the k values of phenolic acids gradually increased with the increase of oxygen concentration. The k values of caffeic acid, chlorogenic acid, theogallin, and ellagic acid in H group increased 84.98%, 104.94%, 45.72%, and 56.71%, respectively, compared with the CK group, which implied sensitivity of phenolic acids on oxygen was different. And linear fit to the oxygen concentration and the k value was also conducted and all R^2^ values were observed to be higher than 0.97. Based on the slope (k_2_) and R^2^ values (Fig. S2 and Table S2), it can be found that oxygen concentration was the limiting factor for the oxidation rate of phenolic acids, and catechins were more sensitive to oxygen concentration than phenolic acids during the fermentation.

Furthermore, the changes in the oxidation rate constants of flavonoid glycosides were not uniform (Fig. 5C). Compared with the L group, the k values of all 11 flavonoid glycosides were increased in CK groups. However, compared with the CK group, the k values of isovitexin and vitexin decreased 19.87% and 1.75% in H group, while the k values of rutin, quercetin diglucoside, quercetin triglucoside, quercetin-3-galactoside, quercetin-3-glucoside, quercetin-3-glucosylrutinosidemyricetin, quercetin-3-galactosylrutinoside, myricetin-3-glucoside, and myricetin 3-galactoside increased by 11.03%, 40.09%, 36.36%, 16.84%, 10.87%, 26.40%, 1.16%, 33.81, and 37.01%, respectively, such results indicated that the sensitivity of flavonoid glycosides to oxygen was more complex than catechins and phenolic acids. In order to elucidate the effect of oxygen concentration on the flavonoid glycoside oxidation kinetics, the linear fit of k value and oxygen concentration was also conducted (Fig. S2 and Table S2). All R^2^ values of the linear fitted curves were higher than 0.9, except for isovitexin, vitexin and quercetin-3-galactosylrutinoside indicating most of flavonoid glycosides oxidation kinetic rates were limited by oxygen concentration. The slope (k_2_) values of myricetin 3-galactoside, myricetin-3-glucoside, quercetin diglucoside, and quercetin triglucoside were significantly higher than those of others representing that the pyrogallol structure was more competitive than the catechol structure during oxidation process. In addition, to explore the sensitivity of different polyphenols to oxygen in relation to their structures, the k and k_2_ values were compared in Fig. S3. It is clear that they had similar variation trend, which indicated the oxidation of polyphenols with high oxidation rate during regular fermentation were more easily affected with changes in oxygen concentration.

## 4 Discussion

**Fig. 6:**
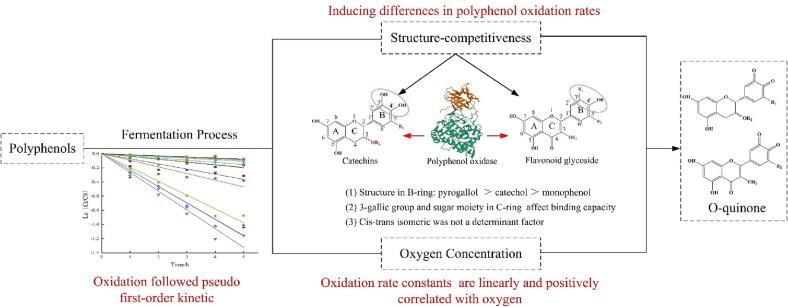
Effect of oxygen and molecular structure on polyphenol oxidation kinetics in black tea fermentation.

Polyphenol oxidation during fermentation is critical for flavor quality formation of black tea (Chen et al., 2021, Wang et al., 2023, Liu et al., 2023). Our results showed that oxidation kinetics of polyphenols, including 7 catechins, 4 phenolic acids and 11 flavonoid glycosides followed pseudo-first-order kinetics during black tea fermentation, while molecular structure and oxygen concentration had differential impacts on polyphenol oxidation. Generally, PPO in tea leaves could first catalyze the oxidation of polyphenols to produce *o*-quinones, and then *o*-quinones and polyphenols could further polymerize with each other to form dimers, trimers and polymers during the black tea fermentation (Verloop et al., 2016, Chen et al., 2021, Xu et al., 2021). Therefore, the catalytic capacity of PPO as well as the competitiveness of the substrates’ structures determinate the oxidation rates of polyphenols. By comparing the oxidation kinetics of polyphenols, pyrogallol structure of polyphenols was found to degrade faster than the catechol or monophenol structure during black tea fermentation (Fig. 6). PPO, an copper-containing enzyme, could catalyze two different reactions including oxidation of the monophenols to o-diphenols and o-diphenols to *o*-quinones, and the monophenolase activity of PPO is usually influenced by the o-diphenolase activity (Ozturk et al., 2016, Koohi et al., 2020). In general, considering the binding ability of the substrate to PPO, o-diphenol is faster than monophenol and the noncatalyzed reaction rate from o-diphenols to *o*-quinones by the atmospheric oxygen oxidation is much faster than that for the oxidation of monophenol to o-diphenols (Hernández-Romero et al., 2010, Kampatsikas and Rompel, 2021). Thus, the catechol structure was oxidized more rapidly than monophenol structure, e.g. quercetin glycosides degraded more rapidly than kaempferol glycosides (Fig. 6). From the electrochemistry point of view, the redox potential is decreased with the increasing amount of hydroxyl groups in polyphenol structures, for example, the redox potential of myricetin (310 mV) with pyrogallol structure was lower than that of quercetin (355 mV) with catechol structure (Choe, Cho, Bae, Yoon, & Lee, 2019). Pyrogallol structure have more hydroxyl groups than catechol structure, and hydrogen bonding between hydroxyl groups of polyphenols and amino residues of PPO is a key driving force for the interaction, and molecular docking simulation also confirmed the pyrogallol structure had general higher docking ability than catechol structure (Xiong et al., 2019, Guo et al., 2021, Song et al., 2021). Besides, catechins with pyrogallol structure are oxidized by a coupling redox reaction with catechol quinones (Narai-Kanayama, Uekusa, Kiuchi, & Nakayama, 2018). Thus, the redox potential of the pyrogallol structure and its ability to bind to PPO makes it more susceptible to be oxidized than catechol structure. In previous work, EGCG and GCG was found to had a stronger ability to inhibit tyrosinase activity by competitive inhibition than other catechins, and the PPO oxidized the non-gallated catechins more efficiently (Wang et al., 2021, Coggon et al., 1973), myricetin glycosides decreased faster than quercetin and kaempferol glycosides (Tan, et al., 2016), and EGC, EGCG decreased obviously faster than EC and ECG in black tea fermentation (Xue, et al., 2022), such observation was further confirmed by our results.

Previously, molecular docking analysis showed that a hydrogen bond could be built between Met280(O) of amino acid residues and C11′-OH(H) of the gallate groups of EGCG (Song, et al., 2021). However, if PPO binds to gallated catechins through the gallate groups, the withdrawing effect of electron could slow the oxidation reaction (Munoz-Munoz, Molina, Molina-Alarcon, Tudela, Garcia-Canovas, & Rodriguez-Lopez, 2008). Thus, gallic acid group in C-ring of catechins was supposed to have inhibitory effect on catechin oxidation (Fig. 6). In addition, PPO dominates the conversion of flavonoid glycosides, and aglycone is the dominant party of the sensitivity of flavonoid glycosides to PPO, while sugar moiety can promote the docking (Guo, et al., 2021). This is consistent with the results of our work, but whether the sugar moiety promotes the oxidation of flavonoid glycosides or not needs to be further verified.

It was reported that the pyrone moiety in B-ring of kaempferol, quercetin and myricetin preferentially chelated copper ions in tyrosinase, also known as PPO, by which the catalytic activity of enzymes on C-ring of flavonols was inhibited but being sustained (Chen and Kubo, 2002, Kubo et al., 2004, Xiong et al., 2019). Thus, it can be observed that the oxidation rate of flavonoid glycosides was slower than catechins during black tea fermentation.

According to the Michaelis-Menten equation, the enzyme oxidation follows first-order kinetics if the amount of enzyme is much higher than that of substrates (Schnell, & Mendoza, 2004). And Kasserra also suggested that, in transient-phase studies, the more initial enzyme amount is required to ensure that the enzymatic reaction follow first-order kinetics (Pettersson, 1976). Thus, it is reasonable to suggest that PPO was sufficient, while polyphenol and oxygen concentration is insufficient in regular black tea fermentation process due to the finding that the oxidation of most polyphenols followed pseudo-first-order kinetics. Interestingly, the k value of oxidation rate constant and oxygen concentration was linearly and positively correlated, which further indicates that oxygen was insufficient during the black tea fermentation. Taken together, providing sufficient oxygen during fermentation could be a promising strategy for promote the quality of black tea products.

## 5 Conclusion

In conclusion, oxidation of the most polyphenols during black tea fermentation followed pseudo-first-order kinetics. Meanwhile, pyrogallol structure of polyphenols in B-ring was oxidized faster than the catechol and monophenol structure, and the gallic group in C-ring of catechins inhibited its oxidation rate, while the specific role of sugar moiety on oxidation of flavonoid glycosides should be further explored. Furthermore, oxygen, not PPO, was suggested to the key factor limiting the oxidation rate of most polyphenol during regular black tea fermentation. Such results indicates that polyphenol oxidation during black tea fermentation depends on their specific molecular structures, which should be taken into consideration for black tea fermentation improvement.

## CRediT authorship contribution statement

**Lin Chen:** Investigation, Data curation, Formal analysis, Software, Writing – original draft. **Huajie Wang:** Writing – review & editing. **Yang Ye:** Conceptualization, Funding acquisition, Writing – review & editing. **Yuefei Wang:** Writing – review & editing. **Ping Xu:** Conceptualization, Resources, Funding acquisition, Writing – review & editing.

## Declaration of Competing Interest

The authors declare that they have no known competing financial interests or personal relationships that could have appeared to influence the work reported in this paper.

## Data Availability

Data will be made available on request.
