# Supplementary material for: Structural insight into polyphenol oxidation during black tea fermentation
Source: Food Chem X. 2023 Feb 26;17:100615. doi: 10.1016/j.fochx.2023.100615 (PMC10039259; doi:10.1016/j.fochx.2023.100615)
Supplement: Supplementary data 1 [file mmc1.docx]

**Supplementary Materials**

**Table list**

**Table S1.** The rate constant and R^2^ of polyphenols degradation.

**Table S2.** The rate constant, standard error and R^2^ value of the liner fitted curves.

**Figure list**

**Figure S1.** Diagram of black tea sample preparation and polyphenols detection.

**Figure S2.** The fitting curves of polyphenols degradation rate constant and oxygen concentration.

**Figure S3.** The polyphenol oxidation rate constant (k) and the slope (k2) value of linear fitted curves between k and oxygen concentration: (A) catechins, (B) phenolic acids, (C) flavonoid glycosides.

**Table S1.** The rate constant and R^2^ of polyphenols degradation.

| **Group** | **Name** | **K** | **R^2^** |
| --- | --- | --- | --- |
| **Catechins** | EC | 0.2131 | 0.98473 |
|  | ECG | 0.15343 | 0.98804 |
|  | EGC | 0.43507 | 0.95239 |
|  | EGCG | 0.25086 | 0.97242 |
|  | C | 0.15802 | 0.96374 |
|  | GC | 0.44657 | 0.94606 |
|  | GCG | 0.49221 | 0.94482 |
| **Phenolic acids** | Caffeic acid | 0.08188 | 0.914 |
|  | Chlorogenic acid | 0.07696 | 0.88805 |
|  | Theogallin | 0.11402 | 0.98356 |
|  | Ellagic acid | 0.07901 | 0.84244 |
| **Flavonoid glycosides** | Isovitexin | 0.07246 | 0.91734 |
|  | Vitexin | 0.0936 | 0.90352 |
|  | Rutin | 0.03999 | 0.91828 |
|  | Quercetin diglucoside | 0.23226 | 0.98557 |
|  | Quercetin triglucoside | 0.26472 | 0.98319 |
|  | Quercetin-3-galactoside | 0.01603 | 0.88692 |
|  | Quercetin-3-glucoside | 0.02161 | 0.94871 |
|  | Quercetin-3-glucosylrutinoside | 0.0308 | 0.92592 |
|  | Quercetin-3-galactosylrutinoside | 0.03863 | 0.86378 |
|  | Myricetin-3-glucoside | 0.23088 | 0.98323 |
|  | Myricetin-3-galactoside | 0.19479 | 0.98807 |

**Table S2.** The rate constant, standard error and R^2^ value of the liner fitted curves.

| **Group** | **Name** | **k_2_** | **standard error** | **R^2^** |
| --- | --- | --- | --- | --- |
| **Catechins** | EC | 0.91323 | 0.04498 | 0.99276 |
|  | ECG | 0.64228 | 0.03762 | 0.98978 |
|  | EGC | 1.64904 | 0.21387 | 0.95118 |
|  | EGCG | 1.00233 | 0.09154 | 0.97539 |
|  | C | 0.76204 | 0.0387 | 0.9923 |
|  | GC | 1.92803 | 0.12657 | 0.98718 |
|  | GCG | 1.92766 | 0.24627 | 0.95258 |
| **Phenolic acids** | Caffeic acid | 0.41787 | 0.02432 | 0.98991 |
|  | Chlorogenic acid | 0.42292 | 0.04076 | 0.97264 |
|  | Theogallin | 0.48876 | 0.03136 | 0.98775 |
|  | Ellagic acid | 0.35871 | 0.00909 | 0.99808 |
| **Flavonoid glycosides** | Isovitexin | 0.2188 | 0.0639 | 0.78144 |
|  | Vitexin | 0.32137 | 0.08167 | 0.82842 |
|  | Rutin | 0.14634 | 0.02493 | 0.91769 |
|  | Quercetin diglucoside | 0.97951 | 0.05759 | 0.9897 |
|  | Quercetin triglucoside | 1.08927 | 0.07275 | 0.98674 |
|  | Quercetin-3-galactoside | 0.06062 | 0.00937 | 0.93165 |
|  | Quercetin-3-glucoside | 0.07665 | 0.01194 | 0.93058 |
|  | Quercetin-3-glucosylrutinoside | 0.12053 | 0.01098 | 0.97549 |
|  | Quercetin-3-galactosylrutinoside | 0.13416 | 0.02965 | 0.86652 |
|  | Myricetin-3-glucoside | 0.94144 | 0.06754 | 0.98472 |
|  | Myricetin-3-galactoside | 0.80506 | 0.05154 | 0.9878 |

**
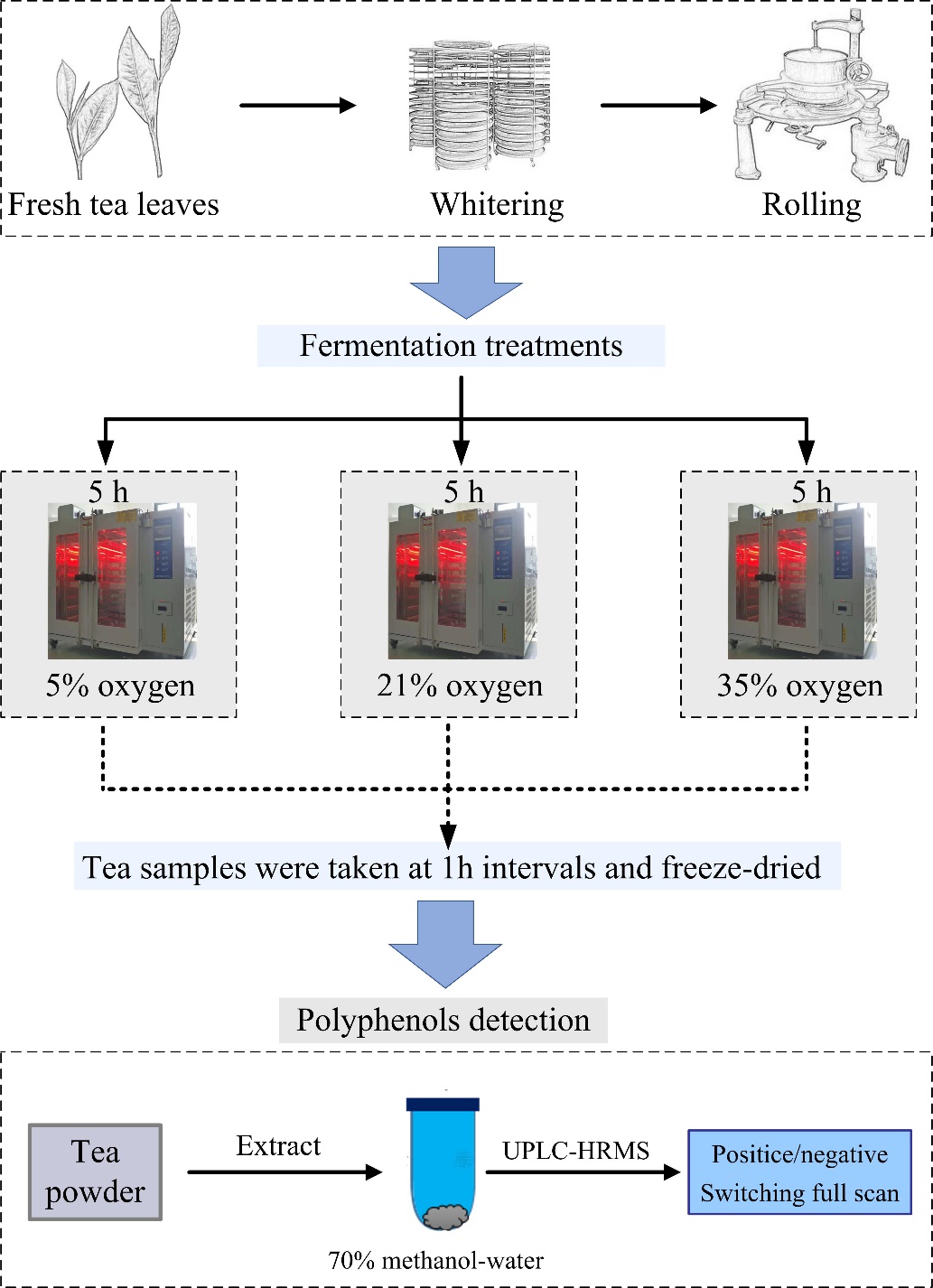
**

**Figure S1.** Diagram of black tea sample preparation and polyphenols detection.


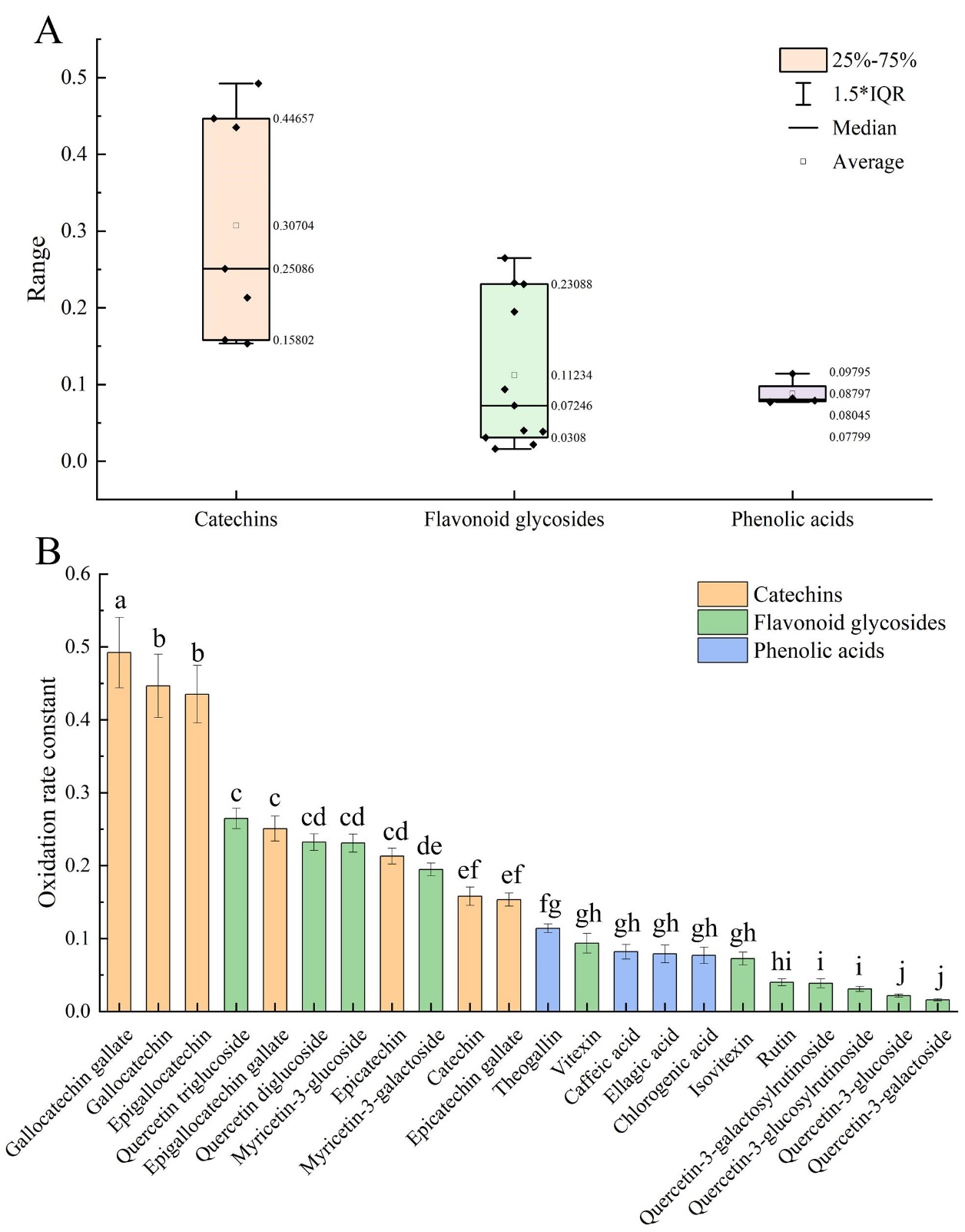


Figure S2. (A) Box plot of degradation rate constants of catechins, flavonoid glycosides and phenolic acids; (B) Oxidation rate constant of catechins, flavonoid glycosides, and phenolic acids.


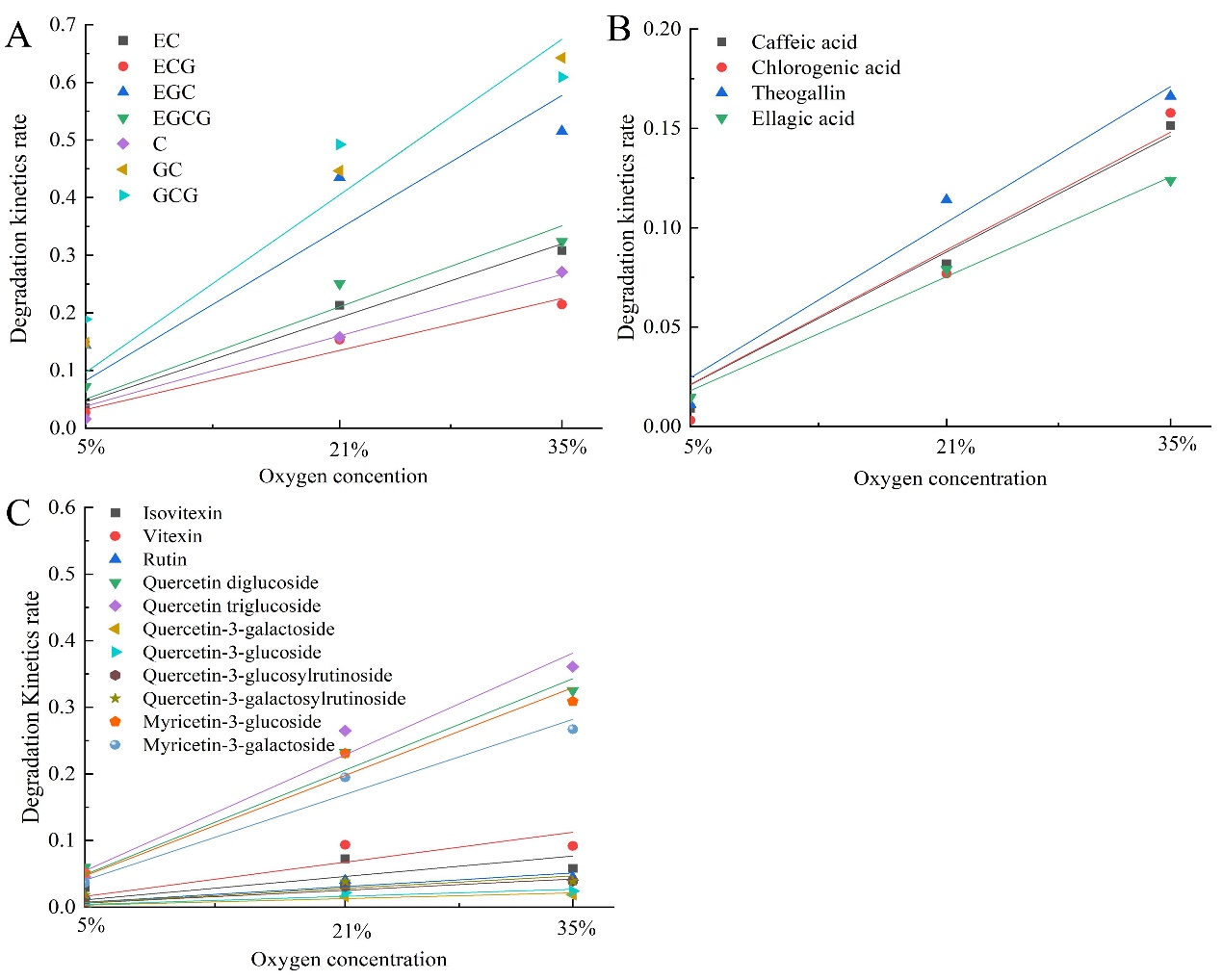


**Figure S3.** The fitting curves of polyphenols degradation rate constant and oxygen concentration.


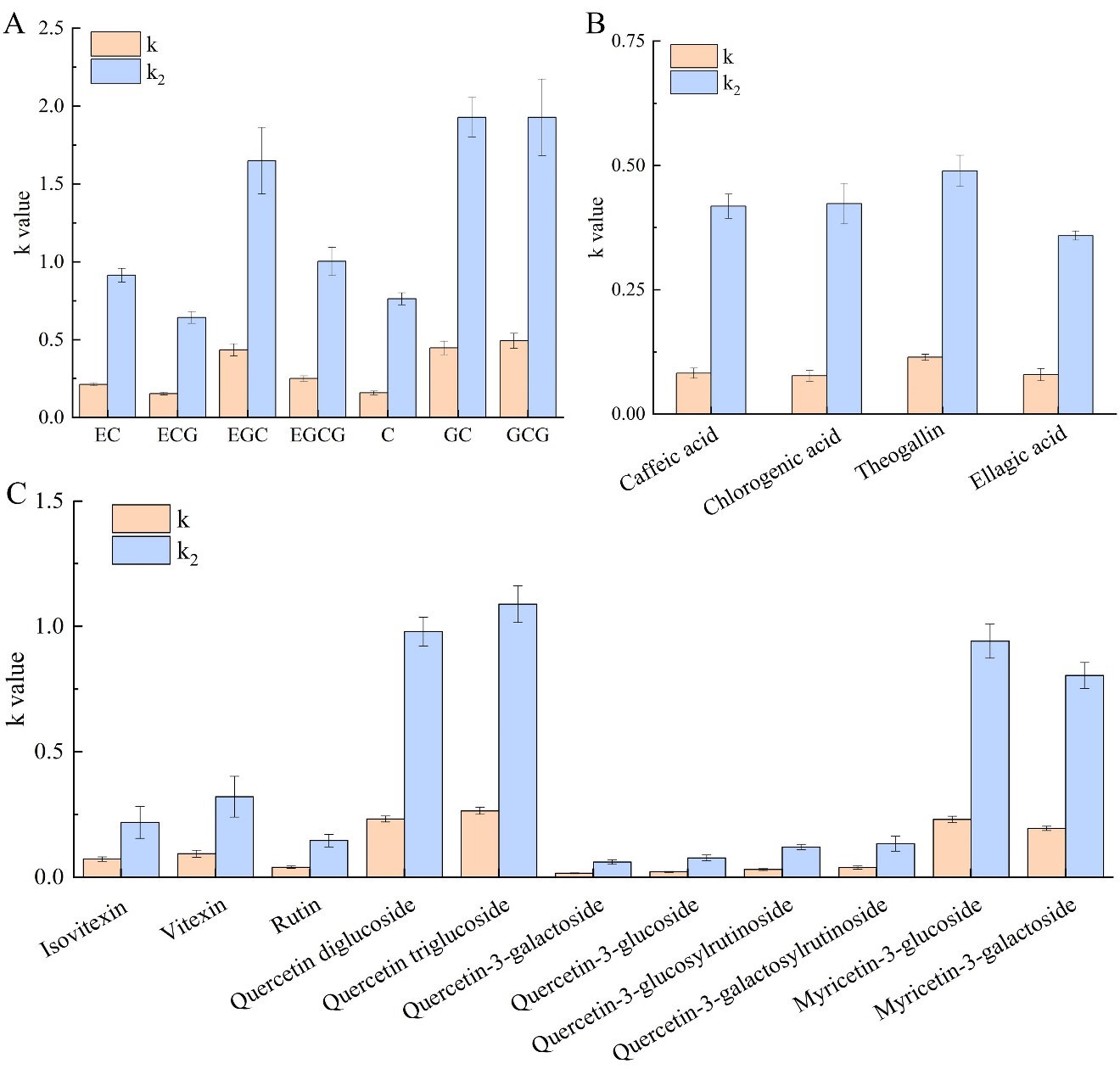


**Figure S4.** The polyphenol oxidation rate constant (k) and the slope (k2) value of linear fitted curves between k and oxygen concentration: (A) catechins, (B) phenolic acids, (C) flavonoid glycosides.
